# Rescuing Flavor Identity and Dynamic Perception in Puréed Dishes; A Restructuring Solution for the Purée Diet

**DOI:** 10.3390/foods10040905

**Published:** 2021-04-20

**Authors:** Elizabeth Carrillo, Laura Laguna, Carla Arancibia, Amparo Tárrega

**Affiliations:** 1 Institute of Agrochemistry and Food Technology (IATA, CSIC), Agustín Escardino, 7, 46980 Paterna, Spain; ecarrillo@iata.csic.es (E.C.); atarrega@iata.csic.es (A.T.); 2 Food Science and Technology Department, Technological Faculty, University of Santiago of Chile (USACH), Technological Obispo Umaña 050, Estación Central 9170201, Chile; carla.arancibia@usach.cl

**Keywords:** purée, flavor, emulsion, identification, structure, Free-TOS

## Abstract

With age, difficulties with masticating and swallowing means food consistency and structure must be modified, such as puréeing food. However, elderly consumers have reported that puréed food’s taste lacks appeal. This study shows how puréeing food changes the recognition and dynamics of flavors and new strategies to improve them. Further, to measure the identification and dynamics of flavor, a new sensory method was investigated that combined Free Choice and Temporal Order of Sensation (Free-TOS). Three dishes (macaroni, pizza, and potato salad), their purées, and three pasta purées with added flavors (cheese and dry-cured ham; added directly or as an oil in water emulsion or using two types of emulsions (oil in water and water in oil) were assessed by three groups of 60 consumers using Free-TOS. Results showed that in the purée the frequency of mentioned sensations decreased compared with the dish, as it was more difficult to identify flavors. Adding flavors in powder form only allowed a cheese/ham flavor identification, but in the purées with emulsions, it was possible to identify the dry-cured ham flavor. Therefore, this study showed that the Free-TOS method does not need a predetermined attribute list and registers the actual identified flavors and their order of appearance.

## 1. Introduction

With age, difficulties in masticating and swallowing require increased food consistency and structure modification. This is done by crushing or mashing food to make dishes (purées) suitable for the elderly population. This constitutes a needed practice in long-term care facilities for elderlies, like nursing and care homes, where they must quickly and easily adapt the food to all residents. However, this food modification has disadvantages: When water is added to form a purée, the nutritional quality is diluted; and there is a loss of the sensory appeal [1]. In fact, when solid food is converted into a purée, consumers, especially the elderly, have reported the food lacks visual, textural, and taste appeal [2], making the food boring and tedious to eat [1]. This leads to lower food intake and increases elderly vulnerability to malnutrition [3].

To avoid the low intake caused by the lack of sensory appeal, researchers have proposed different routes of improvement. For example, using only positive food descriptive adjectives when serving purées to the nursing home residents [4]. To modify the eating atmosphere with music, flavor, and lighting [5]. To shape purées [6] or 3D print them in a food-like form [7]. To include crispy food sounds while eating the purée [8]. To tailor the sensory quality of the dishes to the elderlies’ likes, or to provide more food variety within a meal [9]. However, to the best of our knowledge, there is no research on improving the flavor profile of the food made into a purée.

Our initial hypothesis is that when a dish is transformed into a purée, the dynamics of flavor perception are altered, and the different flavor compounds become mixed. Therefore, individual flavors might be difficult to identify, resulting in a homogeneous and boring food product when eaten. One effective strategy to mitigate the loss of flavor dynamics and create a more desirable sensory experience when eating a purée could be to change the temporality of flavor appearance and intensity. This is possible using different techniques, such as changing the food structure by adding different thickeners [10,11] or adding more flavors. However, this might delay the perception of the flavors (when adding thickeners) or increase the intensity (when adding more flavors), but it will not alter the temporality of the sensations, and all flavors will be perceived at once. A solution might be to individually isolate the different purée flavors, for example by using encapsulation. At an industry level, encapsulation is favorably used to avoid flavor deterioration over time, this is commonly done by coacervation or spray chilling [12]. However, if the release of the flavor in-mouth needs to be simpler, encapsulation techniques can be used, like emulsification, where flavors, depending on their hydrophobicity, will be dispersed in the water or in the fat phase. In-mouth, and due to saliva and oral movements, the emulsion will be destabilized by coalescence or flocculation [13,14], and it is expected that the encapsulated flavor will be released once the food matrix is broken.

To measure the in-mouth flavor sequence of sensations, different dynamic sensory methods can be used, for example by using the Temporal Dominance of Sensations (TDS) or Temporal Check All That Apply (TCATA). TDS is based on the selection, from a list of predetermined terms, of one dominant sensation at each moment during consumption of a food product [15]. In the TCATA, which is a temporal extension of Check All That Apply (CATA), assessors are provided with a predetermined list of attributes and they can select all the terms they consider applicable at each point in the evaluation [16]. This predetermined list could falsely lead assessors to choose a flavor they might not perceive or to not be able to choose a flavor they might perceive as is not on the provided list. Therefore, to study the flavor dynamics and to know which flavors consumers can identify, this study proposes a new method that combines Free Choice (Free) and Temporal Order of Sensations (TOS), and is thus called Free-TOS. The Free-TOS allows each assessor to create their own list of terms (free elicitation) concerning their perception; then, the assessors can order the first three attributes they perceive during tasting and aftertaste, according to their own individual list of attributes.

The objectives of this study were first, to study how puréeing food changes flavor recognition and the sequence of flavor perception. Second, to rescue the flavor recognition, intensity, and sequence by adding flavors directly to the purées or isolated flavors incorporated in different emulsions. Last, to study the feasibility (setting and analysis) of the Free-TOS method.

## 2. Materials and Methods

### 2.1. Comparing Flavor of Dishes and Purées

#### 2.1.1. Materials

Ingredients for preparing dishes and purées were purchased in a local supermarket. For the pasta preparation, ingredients were penne pasta (penne rigate; Hacendado) (38.5% *w*/*w*), tomato sauce with onion (Hacendado) (35%), dry-cured ham dices (Incarlopsa) (17%), and Grana Padano cheese (Zanetti) (9.5%). Ingredients used to prepare the pizza were a pizza base (Hacendado) (39.8%), chicken (Vall Company) (29%), grated mozzarella cheese (Hacendado) (15%), tomato sauce (Hacendado) (16%), oregano (Hacendado) (0.1%), and basil (Hacendado) (0.1%). For the potato salad, the ingredients used were frozen mixed vegetables (potato, carrot, green beans, and green peas; Hacendado) (59%), tuna (Hacendado) (16.5%), mayonnaise (Hacendado) (10%), boiled eggs (7%) (Hacendado), pickled cucumbers (Hacendado) (4%), red bell pepper (3%), and salt (Hacendado) (0.5%).

#### 2.1.2. Dish and Purée Preparation

The three dishes (pasta, pizza, and potato salad) were prepared using a food processor (Thermomix TM5, Wupertal, Germany). The preparation procedure for each dish was:

Pasta: Dry penne pasta was boiled for 10 min; after the water was drained. Dry-cured ham dices were fried for 1 min, then the rest of the ingredients (pasta, tomato sauce, and cheese) were added and mixed at a minimum speed for 3 min in the food processor.

Pizza: Chicken breast was cut in small portions and fried for 3 min. After that, the tomato sauce was spread on the pizza base and the chicken was added, topped with the cheese. The oregano and basil were added on top of the cheese and the pizza was baked for 12 min at 225 °C in a conventional oven.

Potato salad: Frozen mixed vegetables were boiled in salted water for 12 min. Then the water was drained, and the vegetables were refrigerated. When the vegetables were cold, the boiled egg, pickles, and red bell pepper were cut into pieces, added the rest of the ingredients (mayonnaise and tuna), and were mixed with the boiled vegetables.

Purées: To obtain the purée for each dish, the dish was poured into the food processor, and water was added (22–50%) to obtain an adequate modified texture (Fork Mashable Dysphagia Diet or to level 4 according to the International Dysphagia Diet Initiative) as used in nursing and care homes. This was stirred and blended at 5000 rpm for 10 min. The temperature of the purées was kept at 50 °C for pasta and pizza, and at 40 °C for the potato salad.

#### 2.1.3. Evaluation of Dishes’ and Purées’ Flavor Using Free Temporal Order of Sensation (Free-TOS)

Sixty participants (60% women and 40% men, aged between 21 to 60, average 28.8) evaluated the flavor of the dishes and purées in two sessions. Participants did not have experience in sensory analysis and were recruited through emails and poster announcements at the Science park of Valencia University. The only condition to participate in this study was their willingness and availability to assist at two sessions.

All participants first attended the purée evaluation session, and at least two weeks later, the dish evaluation session.

The sample flavors were evaluated using the Free-TOS technique that comprising two parts:

(1) Free generation of flavor descriptors list. A sample (dish or purée) was served to each participant and they were asked to taste and list all the flavors they perceived for the specific sample. The six samples (in two sessions: first purées and then dishes) were served monadically following a balanced design.
(2) Evaluation of the Temporal Order of Sensation. In the same session, the participant was provided with another of each sample, and their individually generated list of flavors for the specific sample. The participant was asked to read the attributes, to eat one spoon of the sample, and to indicate the first three flavors they perceived (first, second, and third in order of sensations), also the flavor they perceived after swallowing the sample (aftertaste). They followed the same procedure with the second spoon of the sample. The six samples (in two sessions: first purées and then dishes) were served monadically following a balanced design.

The pasta, pizza, and their purées were served at 40 °C; the potato salad and its purée were served at room temperature (20 °C). All samples were labeled with a random three-digit code number. Each participant received a portion of 50 g of sample served on a plate, for dishes, and the purées were served in a 100 mL glass. Between sample evaluation, participants were asked to wait for 1 min, and they were provided with unsalted crackers and water for mouth-rinsing.

### 2.2. Improving the Flavor in the Pasta Purée

#### 2.2.1. Ingredients

For emulsion preparation, three emulsifiers were used: rapeseed lecithin (Cargill Texturizing Solutions Deutschland GmbH & Co. KG, Hamburg, Germany), polysorbate (Tween 80, Sigma-Aldrich, Gillingham, UK), and whey protein (concentrate 515NL SMP, Univar, Heerenveen, The Netherlands). Two flavorings were used: cheese flavor (Gouda flavor, OZ-024-705-5, Givaudan Ibérica SA, Sant Celoni, Spain) and ham flavor (Cured Ham flavor, Soteal, Soluciones Técnicas en Alimentación, Madrid, Spain).

#### 2.2.2. Sample Preparation

Four pasta purée samples were prepared, one with no flavor (base purée) and three with flavorings incorporated in the purée three ways: (i) directly (purée with flavorings), (ii) in an O/W emulsion (purée with oil in water (O/W) emulsion), (iii) in two emulsions (O/W + W/O) (purée with two emulsions (O/W + W/O)). For comparison, the components of the emulsion (sunflower oil at 10%, and a mixture of emulsifier: rapeseed lecithin 3.5% *w*/*w*, polysorbate 1.5% *w*/*w*, and whey protein 0.05% *w*/*w*) were included in all purées. The flavoring concentration was also the same in each of the flavored purées: cheese flavoring (0.15% *w*/*w*) and ham flavoring (0.5% *w*/*w*).

Base purée. The pasta purée was prepared as described in Section 2.1.

Flavored purée without emulsion. Ham and cheese flavor powders were directly added to the pasta purée at the end of its preparation and mixed in the food processor for 5 min.

Purée with oil in water (O/W) emulsion. First, the aqueous phase for the emulsion was prepared to disperse each emulsifier (rapeseed lecithin and polysorbate) in water for 50 min at 500 rpm. Then, sunflower oil was added to the aqueous phase while being mixed with a high-speed homogenizer (IKA T18 basic, Ultraturrax, Wertheim, Germany) with an S18N-19G coupling/accessory for 15 min at 9500 rpm. The cheese flavor was added to the oil phase, and the ham flavor was added to the water phase before the emulsification process. This emulsion was added to the pasta purée at the end of its preparation and mixed in the food processor for 5 min.

Purée with two emulsions (O/W + W/O). Following the same procedure described above, an O/W emulsion was prepared with the cheese flavor in the oil phase and the ham flavor added in the water phase. The water-in-oil (W/O) emulsion was elaborated with whey protein as the emulsifier, first dissolved in water by mixing for 15 min at 500 rpm using the Ultraturrax. Then, this water phase was dispersed in the oil (at 25%) using the Ultraturrax for 15 min at 9500 rpm.

#### 2.2.3. Evaluation of Flavored Purées Using Free-TOS

The Free-TOS method was also employed to evaluate the four flavor-modified pasta purées. A second group of 60 participants (63% women and 37% men, aged between 21 to 60, average 32.6), different from the previous task and recruited following the same procedure as for the first panel, attended one session and evaluated the four purées following the same procedure described in Section 2.1.2. The four samples were served at 40 °C, each participant received a portion of 50 g served in a 100 mL glass following a balanced design. Between sample evaluations, participants were asked to wait for 1 min and were provided with unsalted crackers and water for mouth-rinsing.

#### 2.2.4. Sensory Evaluation: Paired Comparison Test

A third group of 60 participants (70% women and 30% men, aged between 21 to 54, average 30.4), recruited following the same procedures described before, evaluated the differences in the intensity of the tomato, ham, and cheese flavors among the four purées (base and flavored purées) using pair comparison. All possible pairs of samples (six) were compared. Each participant conducted the six pairs of comparison tests over two sessions (three comparisons per session). For each paired comparison, each participant received two samples and was asked to taste them and indicate which one had a more intense tomato flavor, then to indicate the samples with the more intense cheese flavor, and finally the same for the ham flavor.

Samples were presented with random three-digit coded numbers at 40 °C in a 100 mL glass. Between sample evaluations, participants were asked to wait for 1 min, and they were provided with unsalted crackers and water for mouth-rinsing. Data collection was conducted using Compusense Cloud (Compusense Inc., Guelph, ON, Canada).

### 2.3. Data Analysis

From the Free-TOS test, the descriptors and order of sensations for each participant were obtained. The elicited descriptors generated by each participant were listed and the frequency of mention was counted for each one. A z-test was used to calculate and compare the overall frequency of mention and the significant differences between each dish and its purée (*p <* 0.05) for each common descriptor with at least 10% of the mentions of the dish or in the purée.

The descriptors that each participant mentioned for the first, second, and third order of sensation, and the aftertaste were listed, and their frequency of mention was counted. This information was used to build the Free-TOS curves. Only descriptors mentioned by at least 10% of the participants were represented in the graphs. To analyze the significant differences at each order of sensation between one descriptor elicited by the dish and their purée, a z-test was calculated.

To compare the frequencies of mention of the flavors between the base purée and the three enriched flavored purées (Section 2.2.), Cochrane’s test and multiple pairwise comparisons using Sheskin’s critical difference were conducted.

From the paired comparison test, d’ was calculated to determine the differences in the intensity of tomato, cheese, and dry-cured ham between the base purée, purée with flavorings, purée with O/W emulsion, and purée with two emulsions. The significance of the differences was determined according to the Thurstone/Wald Model. All analyses were performed using XLSTAT (version 2020, Addinsoft, Paris, France).

## 3. Results

### 3.1. Flavor Recognition and Temporal Order of Perception when Consuming Dishes and Purées

#### 3.1.1. Flavor Recognition

The number of flavors elicited by each participant was on average higher for dishes (4.2, 4.6, and 5.4 for pasta, pizza, and potato salad, respectively) than for purées (3.7, 3.9, and 4.0 for pasta, pizza, and potato salad, respectively). In Table 1, the relevant flavors (frequency of mention ≥ 10%) and the percentage of consumers that mentioned each are shown for the dishes and the purées. In this table, the number of the different descriptors was lower for the dishes (7, 6, and 9 for pasta, pizza, and potato salad, respectively) than for the purées (11, 10, and 14 for pasta, pizza, and potato salad, respectively). The fact that purées elicited fewer descriptors and the greater diversity between them indicates a lower agreement of flavors recognized in the purée than in the dish.

In the pasta dish, tomato, cheese, and dry-cured ham were recognized by most participants (93, 63, and 48% respectively), but were significantly less recognized (*p <* 0.05) in the purée (70, 32, and 12% respectively). In the pasta purée, other flavors like potato, chicken, carrot, and pepper—not ingredients of the dish/purée and not described in the pasta dish—appeared, indicating a lower degree of recognition. This also occurred for the pizza dish and pizza purée. In the pizza dish, descriptors for tomato, oregano, cheese, and chicken were recognized by most participants (88, 87, 68, and 67%, respectively) and were significantly higher (*p <* 0.05) than in the purée. Meanwhile, in the pizza purée, consumers used other descriptors like potato, pepper, white sauce, and carrot, which were not mentioned for the dish. The potato salad also had a higher frequency of mention of descriptors for tuna, potato, mayonnaise, and eggs (92, 78, 65, and 53% vs. 53, 53, 13, and 27%, respectively) in the dish than in the purée. However, when tasting the puréed potato salad, other descriptors appeared that were related to the original ingredients, like olives or vegetables, or to the dish identified as potato salad.

Furthermore, consumers elicited many other different flavors but with lower frequency (<10%). The number of different descriptors was greater for the purées (pasta: 39; pizza: 51; potato salad: 34) than for the dishes (pasta: 31; pizza: 41; potato salad: 25) indicating lower agreement and a lesser degree of recognition when tasting purées.

#### 3.1.2. Temporal Order of Sensations

**Figure 1:**
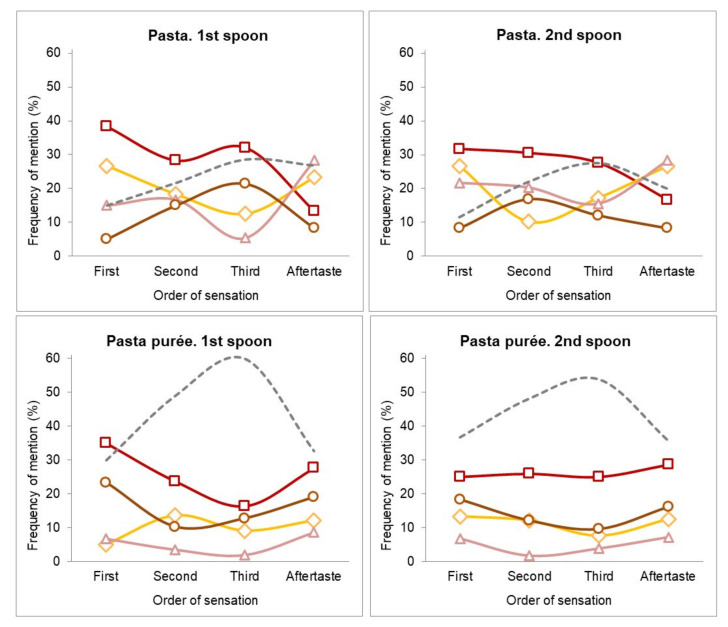
Free-Temporal order of sensation (TOS) plot for the pasta dish and pasta purée. Frequency of mention of the main mentioned flavors: tomato (**□**); cheese (**◊**); dry-cured ham (**∆**); meat (○); and others (**---**) as first, second, and third sensations and aftertaste during the first and second spoon.

Figure 1 shows the TOS perceived when eating the first and second spoon of a pasta dish and its purée. In the pasta dish, the tomato flavor had more mentions in the first, second, and third sensations but decreased in the aftertaste. This indicates it is a relevant flavor perceived during eating but has low persistence after swallowing. Cheese and dry-cured ham were also relevant flavors for pasta, showing a higher frequency of mentions for the first and second sensations and the aftertaste, indicating flavor impact at the beginning of consumption and after swallowing. In contrast, the meat flavor was relevant only as a second and third sensation, indicating that it is a flavor note that appears slowly and is not present after swallowing. For the second spoon, the flavor profile was like the first spoon, but the frequency of mention of the tomato flavor decreased whereas the dry-cured ham flavor became more relevant (higher frequencies of mention).

In the pasta purée, the tomato flavor showed a higher frequency of mention (even in the aftertaste) at all test points, whereas the dry-cured ham flavor showed very few mentions. Meat and cheese showed intermediate mention that changed little over the evaluation.

Comparing purées with their pasta dishes, the frequencies of mention of the main flavors were lower. Like the elicitation task, there was less agreement in the flavors that participants mentioned for each order of perception. Furthermore, as shown in the curve accounting for the other flavors, maximum values in the second and third order indicated less agreement for intermediate stages. For the tomato flavor, frequencies did not significantly change. Furthermore, the cheese flavor, which was relevant as the first sensation in the pasta dish, was not relevant in the purée, with significantly lower frequency (*p <* 0.05). Moreover, the meat flavor was rarely mentioned as the first sensation in the pasta dish but became relevant as the first sensation in the pasta purée with a significantly higher frequency of mention (*p <* 0.05). In addition, the ham flavor was relevant as first and second sensations and in the aftertaste in the pasta dish, but in the purées, the frequencies were significantly lower (*p <* 0.05) with no relevance between the stages.

**Figure 2:**
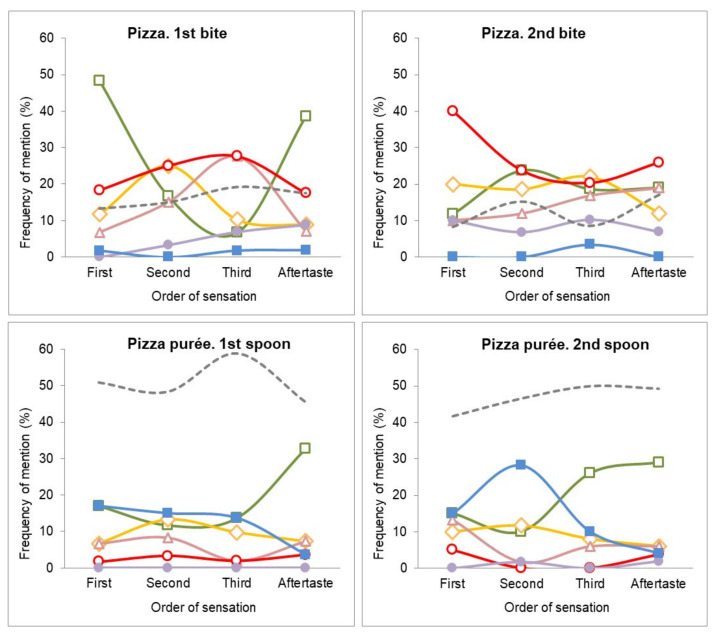
Free-TOS plots for the pizza dish and pizza purée. Frequency of the main mentioned flavors: oregano (**□**); tomato (**○**); chicken (**◊**); potato (**■**); cheese (**Δ**); bread dough (**●**); and others (**---**) as first, second, and third sensations and aftertaste during the first and second spoon.

Figure 2 shows the TOS perceived when eating the first and second bites of the pizza dish and its purée. In the pizza dish, the flavor profiles were different between the first and second bite. Oregano was the most mentioned flavor for the first sensation and aftertaste, for the first bite. It was the first sensation probably because it was on the top of the pizza but decreased during the mastication and mixing when forming a bolus, then the flavor of other ingredients became more relevant (tomato, chicken, and cheese). The second sensation was chicken and tomato with the highest frequency, then cheese and tomato flavors showed the maximum frequency in the third sensation. Later, once swallowed and with no further remaining food in the mouth, the oregano flavor remained the most mentioned flavor. In the second bite, oregano was not the predominant first sensation, indicating an adaptation or habituation to this flavor compound. Tomato and chicken flavors were the most mentioned first sensations, but the frequencies were also high for the second and third sensations. The oregano flavor showed maximum second and third sensation mentions, like the cheese, but for the aftertaste, tomato was still the most mentioned flavor.

In the pizza purée, the frequencies of mentioned flavors were lower than for the pizza dish. The curves were flatter and low agreement between participants mentioning common flavors was observed. The potato flavor appeared as dominant as the first, second, and third sensation, and oregano was dominant in the aftertaste.

Comparing the dish and the purée, oregano and tomato were significantly lower (*p <* 0.05) for the purée at each consumption point and for the aftertaste; however, potato had significantly higher mentions for the purée. Like with the pasta dish, the percentage of “others” mentions was higher in the pizza purée than in the pizza dish.

**Figure 3:**
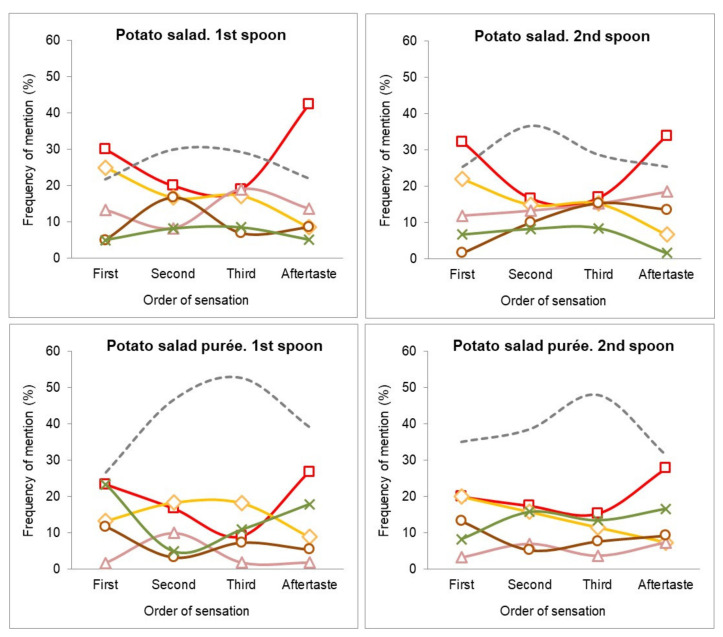
Free-TOS plots for potato salad dish and potato purée. Frequency of main mentioned flavors: tuna (**□**); potato (**◊**); mayonnaise (**Δ**); eggs (**○**); green peas (**x**); and others (**---**) as first, second, or third sensation and aftertaste during the first and second spoon.

Figure 3 shows the TOS perceived when eating the potato salad. For the dish, the first and second spoon had similar profiles, with the tuna and potato flavors being mentioned as first, second, and third sensations. In the aftertaste, the frequency of mention of the tuna flavor was the highest, whereas the potato flavor was not relevant. The egg flavor was relevant as an intermediate sensation (second or third order), and mayonnaise at late stages (third and aftertaste sensations).

For the purée, the dynamic of perception did not differ for the tuna and potato flavor. The mayonnaise flavor was relevant for the first, third, and aftertaste sensations in the potato salad, but in purées showed a significant decrease in the frequencies (*p <* 0.05), becoming non-relevant during its consumption. The green pea flavor that was not relevant in the potato salad became relevant at the first sensation and aftertaste for the potato salad purée (*p <* 0.05).

### 3.2. Impact of Flavoring Strategies on Flavor Perception in Purées.

#### 3.2.1. Flavor Recognition

This section focuses on the four relevant pasta flavors from Section 3.1 (tomato, meat, cheese, and dry-cured ham). Table 2 shows the percentage of consumers in the free elicitation task that identified each flavor descriptor in the base and flavored purées with the three strategies to add the cheese and dry-cured ham flavor (added flavors directly, added flavors in an O/W emulsion, or added flavors in two emulsions O/W + W/O). When adding the flavors, in any form, the frequencies of mention increased for cheese and dry-cured ham in comparison with the base purée. This increase, for the cheese flavor, was only significant when the flavor was added directly (flavored purée without emulsion), and for the dry-cured ham when it was added with one emulsion (O/W emulsion).

#### 3.2.2. Temporal Order of Sensation

**Figure 4:**
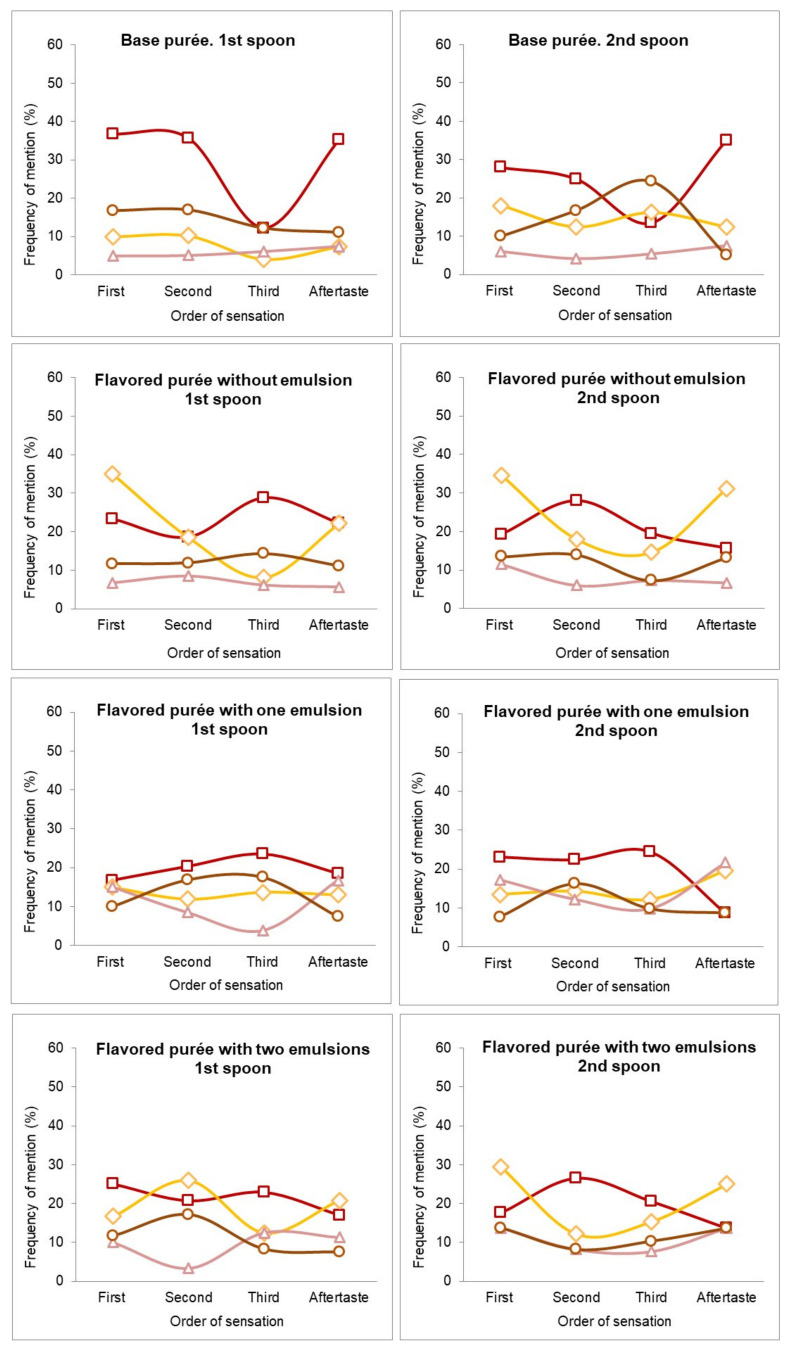
Free-TOS plots for flavor enriched pasta purées. Flavors represented are tomato (**□**); cheese (**◊**); dry-cured ham (**Δ**); and meat (○) as first, second, and third sensations and aftertaste during the first and second spoon.

Figure 4 shows the TOS perceived for the base and the three flavored purées while participants ate the first and second spoons.

For the base purée, the tomato flavor had many mentions as the first, second, and aftertaste sensations with both spoons. The flavor curve for meat was constant for the first and second spoon but decreased for the aftertaste. The cheese flavor is below the meat flavor for the first spoon and slightly increases for the second spoon. Furthermore, the mentions for dry-cured ham were fewer than the other flavors.

In comparison with the base purée, adding cheese and dry-cured ham flavors directly to the pasta purée (flavored purée without emulsion), only enhanced the cheese flavor sensation, which had many mentions as the first order and aftertaste, for both spoons. The tomato flavor profile was also different between the base and the directly added flavorings purée, having an initial lower frequency of mention in the purée with flavorings, as cheese had more frequency of mentions. Then, the tomato flavor overtook the cheese flavor for the second and third order sensations. In the aftertaste, some participants mentioned tomato, and others mentioned cheese. In contrast, for the first spoon, the meat flavor profile was similar in the base purée and in the purée with flavoring. For the second spoon, the meat flavor in the purée with flavorings had a lower frequency of mention in the third order sensation, increasing again in the aftertaste. However, adding flavorings directly to the purée increased the perception of the cheese flavor, but not the dry-cured ham. In previous experiments (data not shown), researchers doubled the concentration of dry-cured ham flavoring and decreased the cheese flavoring, but no changes in perception (identification and temporal order) were observed.

The strategy of adding cheese within the oil phase and dry-cured ham within the water phase of an O/W emulsion in the purée, affected the dry-cured ham flavor, giving and increasing dynamic sensations for the first time for both spoons. Thus, as the cheese flavor was retained, it allowed the perception of the dry-cured ham. In addition, tomato, cheese, or meat were also mentioned as first, second, and third sensations.

Regarding the sensations of the flavored purée with two emulsions (O/W + W/O), the tomato flavor was mentioned less than in the base purée, and the cheese flavor overtook the tomato flavor in the second order and in the aftertaste for both spoons. Furthermore, the dry-cured ham flavor, in the purée with two emulsions, appeared for both spoons.

#### 3.2.3. Flavor Intensity of Purées

**Figure 5:**
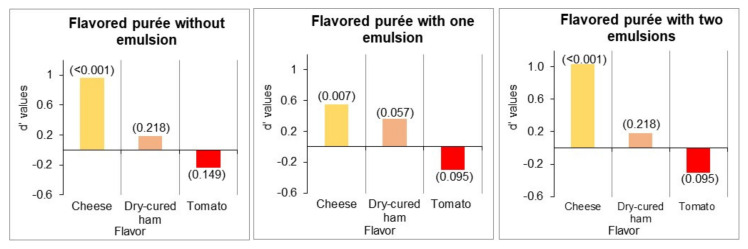
d’ values indicate the differences in the flavor intensity of the three purées with flavorings compared to the base purée. A positive value indicates a more intense flavor, and a negative value indicates a less intense flavor than in the base purée. The value on top of the bars corresponds to the probability indicating the significance of differences.

The differences in the intensity of the cheese, tomato, and dry-cured ham flavors between the base purée and the three purées with added flavorings were determined by calculating d’ values from pair comparison tests (Figure 5). The cheese flavor intensity increased significantly in the three cases, but with a lower effect in the purée with one emulsion. The increase in the intensity of the dry-cured ham flavor was only significant for the purée with O/W emulsion at *p* < 0.10. The intensity of the tomato flavor was slightly lower in the flavored purées, but the difference was not significant. According to these results, only the strategy of flavoring the purée with one emulsion (the O/W emulsion) allowed the increase of both cheese and dry-cured ham flavors.

## 4. Discussion

In this study, the difference in the sensations perceived when eating a dish (pasta, pizza, and potato salad) and when eating two spoons of the same dish in a puréed form was investigated. This comparison, between the dish and puréed dish, agrees with our initial hypothesis, there is a change of flavor dynamics with different food structures (structured dish or purée) and with the initial dish’s degree of structural change. When the food was puréed, the frequency of descriptors mentioned decreases, and new descriptors that corresponded to non-added ingredients in the dish appeared, especially with the pasta and pizza. This is attributed to the lack of agreement of sensations among participants, as the flavor identification was more difficult, and participants guessed more and differently, probably relating to what they think the purée contained or other cues like the starchy texture or appearance. For example, in the pizza or pasta purée, although the purée did not contain potato, potato flavor appeared. The low frequency of flavor mentions, and the change of flavor temporality, was because of the structural rupture of the dishes to convert them into the purée, and the dilution effect because of the water addition to give them the desired texture. Regarding the structure, it has been reported that as flavors are released during the chewing process—and this depends on the food structure [17]—when the food structure is broken down or altered to convert it into a purée, the flavor profile changes. When chewing a food structure, a flavor or flavor combination is released, but if this structure is broken, the combination of flavors is no longer the same, as individual flavors are already mixed, thus leading to loss of their identity. With the potato salad, the change between the dish and the purée was smaller, and there was still a certain dynamic maintained. This might be caused by the initial broken structure of the potato salad, which was presented cut into small cubes, although not puréed, moreover the potato salad was more broken than the pizza or pasta dishes. Because of this smaller change in sensory perception between the dish and purée, less structured dishes (like the cut size of the cubes in the potato salad) when converted into purées, might be better candidates for purées as there is less sensory loss.

Beyond food structure, another variation that can influence flavor perception is oral behavior [18]. It has been reported that different food oral behavior among subjects will influence the sensory perception of foods [19]. However, in this study, although participants chewed freely, and probably because of variation in the chewing patterns, there were differences in the frequency of mention and temporality between dishes and their puréed forms.

Besides the main flavors being less identifiable in the puréed dishes, the change in the frequency of mention between the first and second spoon was less in the purée than in the dish, meaning the sensations between the spoons in the purée were more homogeneous. Previous authors, when studying the sensation evolution over multiple bites, as with the dishes (pasta, pizza, and potato salad evaluation), also found that perceived sensations change between bites or sips [20,21]. The direction (decrease or increase) of a sensation, might depend on the component causing it, and especially how this component interacts with the oral mucosa and saliva. For example, it has been shown that after swallowing wine, an astringency sensation remained, in parallel with the polyphenol contents that caused the astringency sensation [22]. Therefore, depending on which components remained in the oral mucosa, the carry-over effect is different. However, in the puréed dishes with the same components, there is little difference between the first and second spoon. This can happen because consumers could not identify different sensations, as the low frequency of consumers’ agreement shows, and because the food structure was broken, thus food components become mixed and there is a loss of sensory flavor dynamics.

It is clear that the dishes converted into puréed food lose their flavor identity. To address this issue, this study has proposed adding more flavoring directly (powder poured in the purée) and adding the flavors encapsulated in one or two emulsions. To add the cheese flavor as a powder directly to the purée is an easy and quick solution. Further, as this study shows, it can change the dynamic of the flavor profile as well as the intensity, providing more cheese flavor. However, the dry-cured ham flavor was still lost, and it was mainly perceived when cheese was retained in an emulsion form, probably because it was hidden by this flavor. However, when dry-cured ham was added in an emulsion form (purée with two emulsions), the flavor was identifiable, but it had a lower frequency of mention. Therefore, it is convenient to add powerful or more intense flavors in an emulsion form, whereas weaker flavors can be perceived more intensely when dispersed in the continuous phase of the emulsion. Thus, using flavors carried in emulsions, it was possible to modulate the dynamics of perception in puréed dishes. As stated in the introduction, the food industry uses different techniques to encapsulate and protect flavors such as spray drying, spray chilling, spray cooling, extrusion, fluid bed coating, lyophilization, liposomal entrapment, and use microspheres [12]. Furthermore, there are still other encapsulation factors that can also be used in a food industry setting, that can help to tailor the flavor profile of the dish (before purée) such as changing the particle size, flavor load or type, and the concentration of carrier materials [23,24,25,26]. However, in a nursing or care home environment, adding the flavor in an emulsion or to the continuous phase is the most feasible option. Emulsification is a culinary technique already used and is well known in cooking schools when making sauces, batters, or dressings, where whisking is needed to form the emulsion.

This study also showed that the Free-TOS method allows the registering of flavors as they are perceived, the frequency, and the order of appearance. Sometimes the first and second flavor changes between people, but it allows us to know the first, intermediate, and aftertaste flavors perceived. The difficulty found when using the Free-TOS method was in the data analysis, as it produced a large quantity of data from each panelist (different words, different order), making it difficult to visualize the results.

## 5. Conclusions

This study confirms that flavors contained in a puréed dish are less identifiable. Further, when breaking down food structure (puréeing the food), there is a change in the dynamic perception within a spoon and between spoons and in flavor identification. Therefore, to enhance the flavor and its dynamic in a puréed dish, different strategies can be used, such as the addition of flavors in an emulsion which provided a better sensory profile. This approach (flavor in emulsion) is proposed to improve the sensory quality and food enjoyment of those who need to purée their food.

This study also presented a new sensory tool, Free-TOS, which avoids leading sensations perceived, as consumers generated their own vocabulary list to assess a food’s sensory dynamics.

In the future, the study of the acceptability of these purees with the targeted population (elderly) and the study of emulsions broken down during the oral processing when incorporated into a semisolid food will be conducted.

## Figures and Tables

**Table 1:** Frequency of flavors descriptor elicited when tasting the dish and the purée.

		Frequency of Citation (%)	
Dish	Descriptor	Dish	Purée
Pasta	Tomato *	93	70
	Cheese *	63	32
	Dry-cured ham *	48	12
	Meat	47	40
	Pasta *	33	2
	Onion *	28	12
	Saltiness	18	22
	Potato *	0	17
	Chicken *	0	17
	Carrot *	0	17
	Pepper *	0	10
Pizza	Tomato *	88	13
	Oregano *	87	48
	Cheese *	68	25
	Chicken *	67	33
	Bread dough *	38	2
	Onion *	25	15
	Potato *	0	57
	Pepper *	0	17
	White sauce *	0	15
	Carrot *	0	13
Potato salad	Tuna *	92	53
	Potato *	78	53
	Mayonnaise *	65	13
	Eggs *	53	27
	Green peas	50	47
	Carrot	43	42
	Pickle *	42	5
	Red bell pepper	38	28
	Green beans	20	8
	Onion *	0	20
	Potato salad *	0	13
	Olive *	3	12
	Tomato *	0	10
	Vegetables *	0	10

* Indicates that frequency significantly differs between each dish and its purée according to the z-test (*p <* 0.05).

**Table 2:** Percentage of consumers that identified each one of the relevant flavors (tomato, cheese, dry-cured ham, and meat) in the different pasta purées in the free elicitation task.

Descriptor	Base Purée	Flavored Purée without Emulsion	Flavored Purée with One Emulsion	Flavored Purée with Two Emulsions
Tomato	72	73	67	77
Meat	47	45	42	43
Cheese	37 ^a^	65 ^b^	50 ^ab^	55 ^ab^
Dry-cured ham	15 ^a^	22 ^ab^	32 ^b^	25 ^ab^

A different uppercase letter in a row indicates a significant difference between samples according to Cochran’s Q test (*p <* 0.05) and multiple pairwise comparisons using the critical difference (Sheskin’s) procedure.

## Data Availability

Data available on request due to privacy restrictions.
